# PSCA is a target of chimeric antigen receptor T cells in gastric cancer

**DOI:** 10.1186/s40364-020-0183-x

**Published:** 2020-01-28

**Authors:** Di Wu, Jiang Lv, Ruocong Zhao, Zhiping Wu, Diwei Zheng, Jingxuan Shi, Simiao Lin, Suna Wang, Qiting Wu, Youguo Long, Peng Li, Yao Yao

**Affiliations:** 1 School of Life Sciences, University of Science and Technology of China, Hefei, 230027 China; 2 Key Laboratory of Regenerative Biology, South China Institute for Stem Cell Biology and Regenerative Medicine, Guangzhou Institutes of Biomedicine and Health, Chinese Academy of Sciences, Guangzhou, 510530 China; 3 Guangdong Provincial Key Laboratory of Stem Cell and Regenerative Medicine, South China Institute for Stem Cell Biology and Regenerative Medicine, Guangzhou Institutes of Biomedicine and Health, Chinese Academy of Sciences, Guangzhou, 510530 China; 4 University of Chinese Academy of Sciences, Shijingshan District, Beijing, 100049 China; 5 Institute of Hematology, Medical College, Jinan University, Guangzhou, 510632 China; 6 Hefei Institute of Stem Cell and Regenerative Medicine, Guangzhou Institutes of Biomedicine and Health, Chinese Academy of Sciences, Guangzhou, 510530 China

**Keywords:** Chimeric antigen receptor T cells, Gastric cancer, Prostate stem cell antigen, Immunotherapy

## Abstract

**Background:**

Gastric cancer is a deadly malignancy and is a prognostically unfavorable entity with restricted therapeutic strategies available. Prostate stem cell antigen (PSCA) is a glycosylphosphatidylinositol (GPI)-anchored cell surface protein widely expressed in bladder, prostate, and pancreatic cancers. Existing studies have thoroughly recognized the availability of utilizing anti-PSCA CAR-T cells in the treatment of metastatic prostate cancer and non-small-cell lung cancer. However, no previous study has investigated the feasibility of using anti-PSCA CAR-T cells to treat gastric cancer, irrespective of the proven expression of PSCA on the gastric cancer cell surface.

**Methods:**

We determined the expression of PSCA in several primary tumor tissues and constructed third-generation anti-PSCA CAR-T cells. We then incubated anti-PSCA CAR-T cells and GFP-T cells with target tumor cell lines at E:T ratios of 2:1, 1:1, 1:2, and 1:4 to evaluate the therapeutic efficacy of anti-PSCA CAR-T cells in vitro. We also assayed canonical T cell activation markers after coculturing anti-PSCA CAR-T cells with target cell lines by flow cytometry. The detection of a functional cytokine profile was carried out via enzyme-linked immunosorbent assays. We then evaluated the antitumor activity of anti-PSCA CAR-T cells in vivo by establishing two different xenograft GC mouse models.

**Results:**

Anti-PSCA CAR-T cells exhibited upregulated activation markers and increased cytokine production profiles related to T cell cytotoxicity in an antigen-dependent manner. Moreover, anti-PSCA CAR-T cells exhibited robust anti-tumor cytotoxicity in vitro. Importantly, we demonstrated that anti-PSCA CAR-T cells delivered by peritumoral injection successfully stunted tumor progression in vivo. However, intravenous administration of anti-PSCA CAR-T cells failed to reveal any therapeutic improvements.

**Conclusions:**

Our findings corroborated the feasibility of anti-PSCA CAR-T cells and their efficacy against gastric cancer, implicating the potential of applying anti-PSCA CAR-T cells to treat GC patients in the clinic.

## Introduction

Gastric cancer is a significant public health issue, as the fourth most common cancer and the third leading cause of cancer death worldwide [1]. The overall patient survival is significantly poor because of late diagnosis and suboptimal therapies. For early diagnosed patients, combinations of chemotherapy, radiation therapy, or target therapy may be recommended, and many cases can be cure [2]. Unfortunately, the disease is usually diagnosed in an advanced stage, with a reduced response rate to preceding therapies, which underscores the urgency of discovering new treatment modalities.

Chimeric antigen receptors (CARs) are genetically modified receptors that redirect T cells to various tumor surface antigens [3]. First generated in the late 1980s and later developed in the early 2010s, a CAR molecule now generally contains an extracellular antigen-binding domain, an intracellular signaling domain, and one or two additional intracellular costimulatory signaling domains [4, 5]. For clinical use, T cells are harvested from either patients or healthy donors, manipulated to express a specific receptor protein, and then infused into patients after expansion. Acting as a living drug, CAR-T cell therapy is rapidly emerging as a promising new treatment for hematological and nonhematological malignancies [6]. In 2017, the FDA approved Novartis’s tisagenlecleucel for pediatric B cell precursor acute lymphoblastic leukemia (ALL) and Kite’s axicabtagene ciloleucel for adult diffuse large B cell lymphoma [7, 8], thus pushing forward the development of CAR-T cell therapy. Interests have been increased around the possibility that similar success could be achieved in solid tumors. A growing number of preclinical and clinical trials have been conducted ever since [9–11]. Despite the characterization of EpCAM and claudin as new target antigens in gastric cancer [12, 13], disease heterogeneity remains a substantial obstacle for solid tumor immunotherapy [14], necessitating the identification of alternative antigens.

PSCA, formerly denoted as prostate stem cell antigen, is a glycosylphosphatidylinositol (GPI)-anchored cell surface protein belonging to the Thy-1/Ly-6 family. This antigen has been recognized as a critical marker in several primary cancers, including bladder, prostate, and pancreatic cancers [15]. Existing studies have thoroughly acknowledged the availability of utilizing anti-PSCA CAR-T cells in the treatment of metastatic prostate cancer and non-small-cell lung cancer [16, 17]. Specifically, a phase I study employing anti-PSCA CAR-T cells for the treatment of patients with metastatic castration-resistant prostate cancer has recently been initiated. Although extensive research applying this target in multiple malignancies has been carried out, no single study has verified the applicability of targeting PSCA in gastric cancer, irrespective of its proven expression on the gastric cancer cell surface [18].

To address the feasibility of using anti-PSCA CAR-T cells to treat GC, we first confirmed PSCA expression in numerous primary GC tissues and multiple cell lines. T cells encoding a PSCA-specific CAR exhibited upregulated activation markers and secreted abundant cytokines critical for T cell immunity upon coculture with BGC-823 cells. Anti-PSCA CAR-T cells also exerted increasing cytotoxicity from low to high E:T ratios. In particular, we showed that the peritumoral application of anti-PSCA CAR-T cells in xenograft GC mouse models imposed efficient tumor control. Altogether, we provided proof-of-principle data for the use of anti-PSCA CAR-T cells in the treatment of gastric cancer.

## Methods

### Generation of the anti-PSCA lentiviral vector

The anti-PSCA scFv fragment was derived from the humanized IG8 anti-PSCA antibody [19] and was synthesized by Genscript Co., Ltd. (NanJing, China) after codon optimization. This fragment was then cloned into a previously reported lentiviral vector containing both CD28 and DAP10 intracellular domains.

### Lentivirus production

To produce lentivirus particles, 293 T cells cultured in DMEM (Gibco, Life Technologies) were co-transfected with the aforementioned anti-PSCA plasmid together with the packaging constructs psPAX2 and pMD2g using polyethyleneimine (Sigma-Aldrich, St Louis, MO, USA). The supernatant was collected 24 h, 48 h, and 72 h post-transfection and filtered through a 0.45 μm filter.

### Generation and expansion of CAR-T cells

PBMCs were extracted from whole blood obtained from healthy donors through Ficoll-Hypaque density gradient centrifugation (Fresenius Kabi Norge, AS, Berg i Østfold, Norway), while primary human T cells were isolated from PBMCs by means of negative selection with the Pan-T Isolation Kit (Miltenyi Biotec, Germany) and activated by microbeads coated with anti-human CD3, anti-human CD2, and anti-human CD28 antibodies (Miltenyi Biotec, Germany) at a 1:1 ratio for 48 h. Afterwards, T cells were transfected with 293 T cell supernatant containing lentivirus particles for 6–8 h in the presence of 8 μg/ml polybrene (Sigma). Two rounds of transduction were conducted, after which T cells were maintained in RPMI-1640 medium supplemented with 10% heat-inactivated fetal bovine serum (FBS), 300 IU/ml interleukin-2 (IL-2), and 1% penicillin/streptomycin.

Peripheral blood mononuclear cells (PBMCs) were generated from healthy donors following informed consent and approved by the Research Ethics Board of Guangzhou Institutes of Biomedicine and Health (GIBH).

### Cell lines and reagents

293 T cells were used for lentivirus production and were maintained in DMEM (Gibco, Life Technologies) supplemented with 10% fetal bovine serum (FBS), 2 mM l-glutamine, 50 μM β-mercaptoethanol, 100 IU/ml penicillin, and 100 IU/ml streptomycin. BGC-823 (human gastric adenocarcinoma), KATO III (human gastric carcinoma), and MKN-28 (human gastric carcinoma) cell lines were obtained from IBCB (Shanghai, China) and were cultured in RPMI-1640 complete medium. These cell lines were then transduced with a lentivirus vector coexpressing green fluorescent protein (GFP) and luciferase to generate GL-labeled cells.

### Flow cytometry

Cells were harvested and suspended in 50 μl 1 x PBS and primary labeled antibodies were added subsequently. The antibodies used included anti-human PSCA-APC antibody (clone 75) from Santa Cruz Biotechnology (Dallas, TX, USA), anti-human CCR7-APC (clone 3D12), anti-human CD62L-PE (clone DREG-56), anti-human CD45RA-APC (clone HI100), anti-human CD45RO-PE (clone UCHL1), anti-human CD27-PE (clone M-T271), anti-human CD25-PE (clone BC96), anti-human CD69-APC (clone FN50), anti-human CD107a-APC (clone H4A3), anti-human CD3-APC (clone UCHT1), anti-human CD4-PerCP/Cy5.5 (clone OKT4), anti-human CD8-PE (clone OKT8), mouse IgG2a isotype control-APC (clone RMG2a-62), and mouse IgG1 kappa isotype control-PE (Biolegend, San Diego, CA, USA). The samples were incubated at 4 °C for 30 min before analyzed by NovoCyteTM (ACEA Biosciences), and data were analyzed using FlowJo software (FlowJo, LLC, Ashland, OR, USA).

### Cytotoxicity assays

The cytolytic activity of anti-PSCA CAR-T cells against BGC-823-GL, KATO III-GL, and MKN-28-GL cells was evaluated by incubating the target cells with anti-PSCA-expressing T cells at the indicated ratio in triplicate wells of white 96-well plates. Target cell viability was monitored 24 h later by adding 100 μl/well D-luciferin (potassium salt) (Yeasen, China) at 100 μg/ml. The background luminescence was negligible (< 1% of the signal from wells containing only target cells). The percentage viability was calculated with the following formula: experimental signal/maximal signal × 100, and the percentage of lysis were normalized to 100% viability.

### Cytokine release assays

Enzyme-linked immunosorbent assay (ELISA) kits for IL-2, interferon-γ (IFN-γ), TNFα, and granulocyte-macrophage colony-stimulating factor (GM-CSF) were obtained from eBioscience (San Diego, CA, USA), and all ELISAs were performed following the operation manual. T cells were co-cultured with target cells at an E:T ratio of 1:2 for 24 h. The culture supernatant was then collected and analyzed for the secretion of IL-2, IFN-γ, GM-CSF, and TNFα by using ELISA kits.

### CDX models for CAR-T cell treatment

Animal experiments were performed in the Laboratory Animal Center of the Guangzhou Institutes of Biomedicine and Health (GIBH). All procedures were undertaken under the approval of the Institutional Animal Care and Use Committee of GIBH. NOD-SCID-IL2Rg−/−mice were generated as previously described [20]. To develop the cancer xenograft models, NSI mice aged from 6 to 10 weeks were used. All mice were maintained in specific pathogen-free (SPF)-grade cages and provided with autoclaved food and water.

For the BGC-823 cell line-based GC subcutaneous (s.c.) xenograft models, 5*10^5^ tumor cells suspended in 150 μl PBS were injected subcutaneously into the left flanks of NSI mice. When the tumor nodules were palpable, the mice were treated with 2.5*10^6^ GFP-T cells or anti-PSCA CAR-T cells both by intravenous injection and peritumoral injection. Tumor volume was measured twice a week and was on the track record for over 3 weeks by a vernier caliper. For the MKN-28 subcutaneously transplanted xenograft models, 2.5*10^6^ tumor cells were injected, and T cells were delivered at a more challenging time point when tumor size went up to around 100 mm^3^, except which other procedures were followed by the same instructions as described above.

### Statistics

Statistical analysis was performed using GraphPad Prism, Version 7(GraphPad, Inc., San Diego, CA, USA); *p* values were calculated by unpaired t-test, * indicates *p* < 0.05, ** indicates *p* < 0.01, and *** indicates *p* < 0.001.

## Results

### PSCA expression in patient tissues and gastric cancer cell lines

**Fig. 1:**
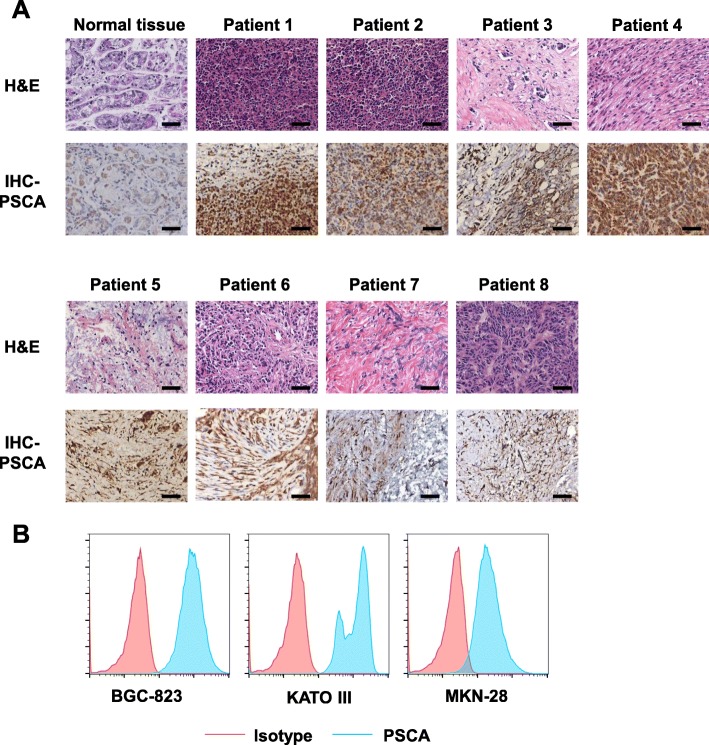
Prostate stem cell antigen (PSCA) expression in primary GC tissues and cell lines. a. Immunohistochemical staining for PSCA in normal gastric tissue and eight primary GC samples; scale bar = 100 μm. b. Detection of PSCA expression in three human GC cell lines, BGC-823, KATO III, and MKN-28 cells, by flow cytometry

To evaluate the potential of the tumor antigen PSCA as an immunotherapeutic target, we immunohistochemically detected its presence and abundance in eight primary gastric cancer samples (Fig. 1a). Most of the analyzed gastric cancer samples expressed PSCA at various frequencies compared to normal tissues. We also performed flow cytometry in several gastric cancer cell lines. The cell types employed in this experiment included BGC-823, MKN-28, and KATO III cells. Uniform expression of PSCA was detected on the surface of these cells (Fig. 1b). Altogether, these data revealed PSCA as a possible novel target for CAR-T cell therapy in GC.

### Generation and characterization of anti-PSCA CAR-T cells

**Fig. 2:**
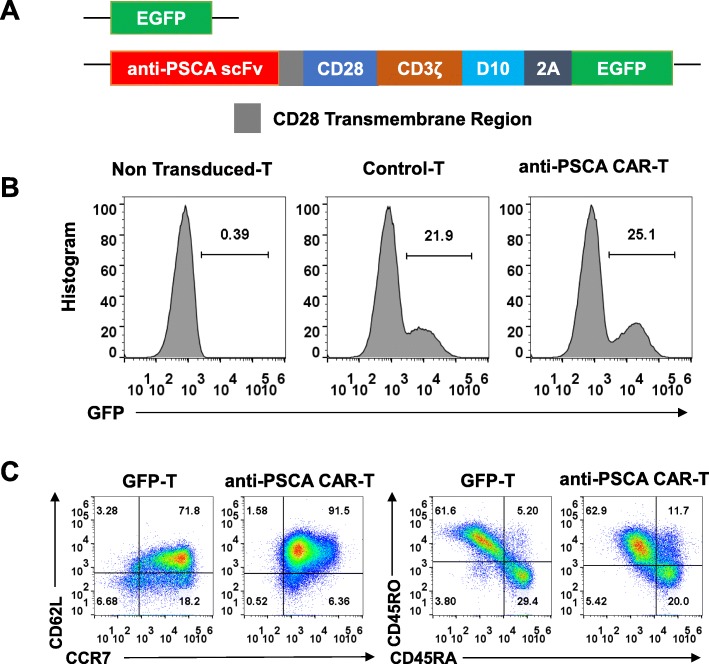
Generation of anti-prostate stem cell antigen (PSCA) CAR-T cells. **a**. The discrete CAR units of anti-PSCA CAR-T cells and GFP-T cells. **b**. Representative flow cytometric analyses of transfected T cells detected by flow cytometry. **c**. CCR7, CD62L, CD45RA, and CD45RO expression was detected on T cells after their generation

We then constructed a third-generation CAR using a humanized single-chain variable fragment (scFv) derived from a mouse anti-human PSCA antibody and a third-generation lentivirus vector composed of a CD3ζ intracellular domain and two costimulatory domains, those of CD28 and DAP10, as previously described [20] (Fig. 2a). T cells transfected with only enhanced green fluorescent protein (eGFP) served as the control for unspecific tonic CAR signaling. Primary human T cells were isolated from peripheral blood mononuclear cells (PBMCs) by magnetic selection and were then activated for 48 h using CD3/CD28/CD2 beads. CAR expression was detected 48 h after lentivirus transduction by flow cytometry according to the GFP-positive proportion (Fig. 2b). Transduced T cells were cultured for 10 days and achieved a final CD45RO^**+**^CCR7^**+**^CD62L^**high**^ phenotype (Fig. 2c), implicating their presumed sustainable antitumor potential in vivo.

### Anti-PSCA CAR-T cells exhibited potent cytotoxicity against GC cell lines

**Fig. 3:**
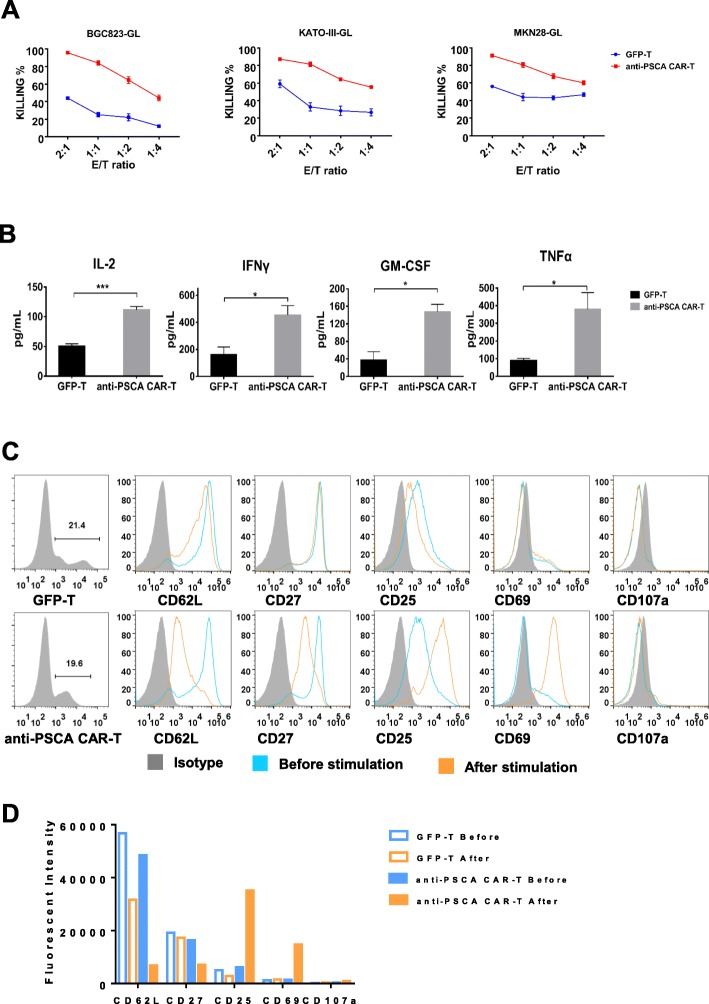
Anti-PSCA CAR-T cells exhibited dramatic antitumor efficacy ex vivo. **a**. The lytic capacity towards different target cells, including BGC-823, KATO III and MKN-28 cells, was analyzed at the indicated effector-to-target ratios in a 24 h lysis assay. **b**. The concentrations of IL-2, IFN-γ, GM-CSF, and TNFα released by anti-PSCA CAR-T cells and GFP-T cells after coculture with BGC-823 cells overnight at an E:T ratio of 1:1 are shown. Error bars denote the s.e.m., and the results were calculated by an unpaired t test. * indicates *p* < 0.05; ** indicates *p* < 0.01; and *** indicates *p* < 0.001. **c**. Canonical T cell markers were detected by flow cytometry at a recommended E:T ratio of 1:1 after coculturing anti-PSCA CAR-T cells and GFP- T cells with target cell lines. **d**. Statistical analysis of three independent FACS results. Error bars denote the s.e.m.

Next, we sought to evaluate the therapeutic efficacy of anti-PSCA CAR-T cells in vitro. To determine the cytotoxicity of transduced T cells in a more delicate and sensitive way, we genetically modified three target cell lines, BGC-823, MKN-28, and KATO III, to express GFP-luciferase. Thus, cell viability could be determined by a luciferase reporter system and an illuminator [21]. We then incubated anti-PSCA CAR-T cells and GFP-T cells with the aforementioned target tumor cell lines at E:T ratios of 2:1, 1:1, 1:2, and 1:4. The results showed that anti-PSCA CAR-T cells exhibited more robust cytotoxicity than GFP-T cells after incubation for 24 h (Fig. 3a). To further investigate the cytokine secretion profile of anti-PSCA CAR-T cells in response to target tumor cells, we collected the culture supernatant from the killing assay described above, and the secreted cytokines were quantified via enzyme-linked immunosorbent assay (ELISA). Cytokines, including interferon-γ (IFN-γ), IL-2, granulocyte-macrophage colony-stimulating factor (GM-CSF), and TNFα, which are generally secreted by activated T cells, were examined (Fig. 3b). It was unsurprising to see that anti-PSCA CAR-T cells produced significantly more functional cytokines than control GFP-T cells.

To determine whether anti-PSCA CAR-T cells could be activated by antigen-expressing tumor cells in a target-specific manner, we cultured anti-PSCA CAR-T cells and GFP-T cells with BGC-823 tumor cells at a 1:1 ratio. It has been reported that T cell activation and differentiation can be monitored based on changes in the expression of a series of surface biomarkers [20]. After coculture with target cells for 24 h, a T cell surface marker profile was detected by flow cytometry. It was demonstrated that anti-PSCA CAR-T cells were fully activated after coculture with target tumor cells, as indicated by their upregulation of CD25 and CD69 (Fig. 3c, d). Activated anti-PSCA CAR-T cells also downregulated CD62L (Fig. 3c, d), a “homing receptor” that is highly expressed on central memory T lymphocytes after they encounter antigen but is not found on effector memory T lymphocytes. Moreover, CD27, a costimulatory immune checkpoint molecule, was also downregulated on anti-PSCA CAR-T cells (Fig. 3c, d). Collectively, these experiments demonstrated that CAR-T cells targeting PSCA were fully activated after encountering target tumor cells and exhibited robust cytotoxicity by producing traditional cytokines related to T cell cytotoxicity.

### Anti-PSCA CAR-T cells showed productive antitumor activity in vivo

**Fig. 4:**
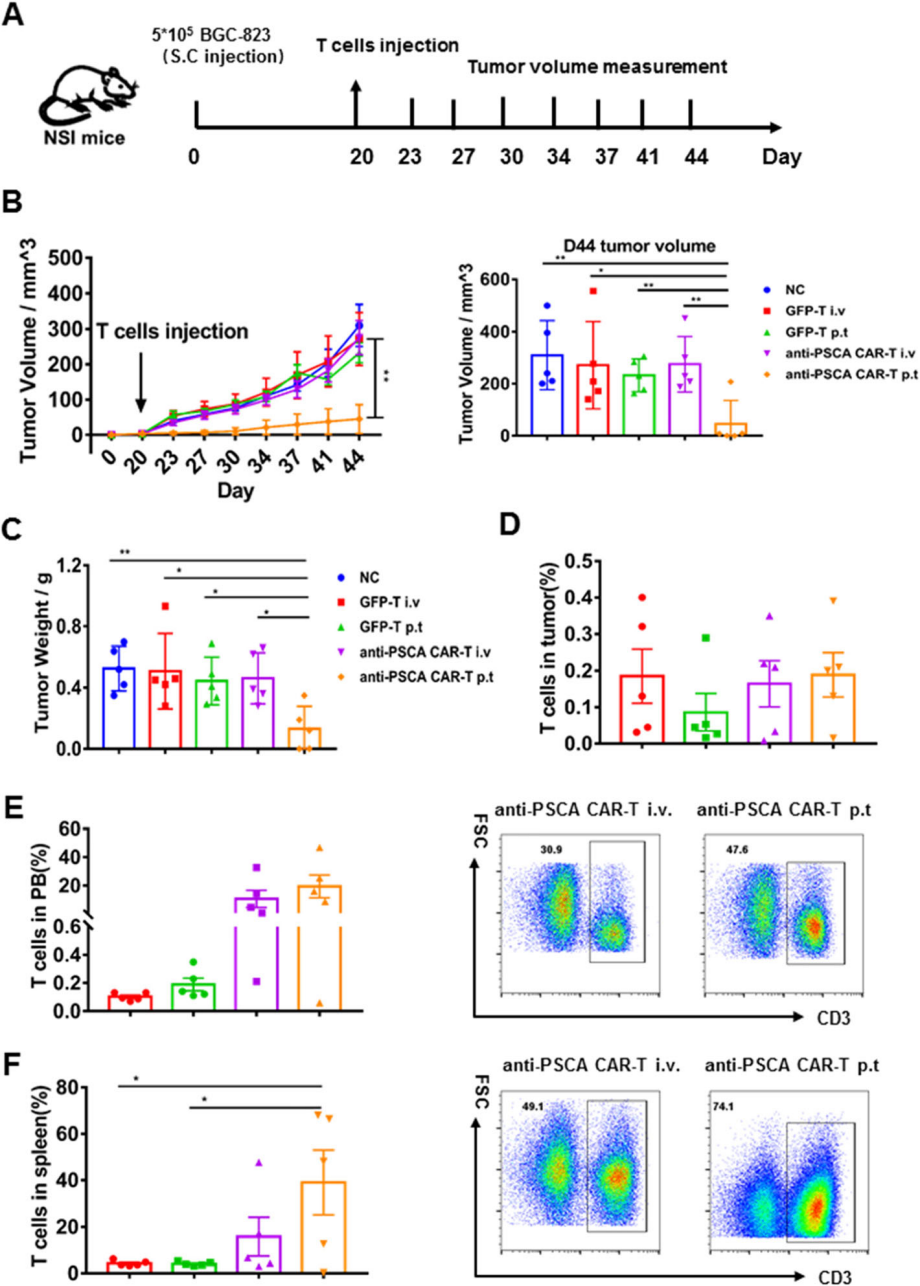
Anti-PSCA CAR-T cells efficiently reduced tumor progression in BGC-823 models. **a**. Schematic representation depicting the time course of the experiment. **b**. Tumor volume was calculated according to the following formula: length × width^2^/2. **c**. Tumor weight of BGC-823 subcutaneously injected mice. Error bars denote the s.e.m., and the results were compared with one-way ANOVA. *p < 0.05; **p < 0.01; ***p < 0.001. **d**. Percentage of T cells in the tumor of the BGC-823 models. **e**. Percentage of T cells in the PB of the BGC-823 models and representative FACS plots of the i.v group and the p.t group, respectively. **f**. Percentage of T cells in the spleen of the BGC-823 models and representative FACS plots of the i.v group and the p.t group, respectively. Error bars denote the s.e.m., and the results were compared with one-way ANOVA. *p < 0.05; **p < 0.01; ***p < 0.001

**Fig. 5:**
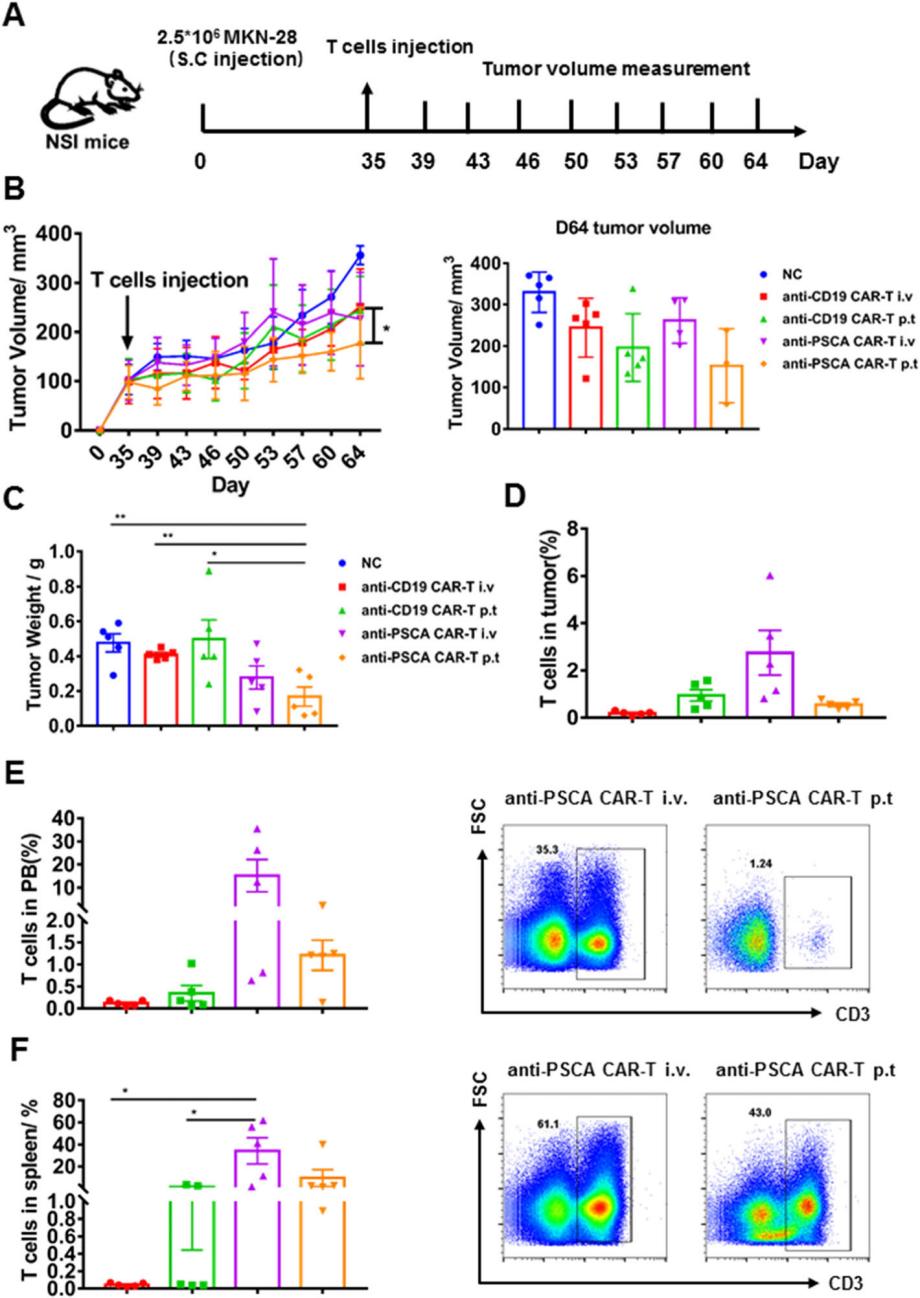
Anti-PSCA CAR-T cells suppressed tumor progression in MKN-28 models. **a**. Schematic representation depicting the time course of the experiment. **b**. Tumor volume was calculated according to the following formula: length × width^2^/2. **c**. Tumor weight of MKN-28 subcutaneously injected mice. Error bars denote the s.e.m., and the results were compared with one-way ANOVA. *p < 0.05; **p < 0.01; ***p < 0.001. **d**. Percentage of T cells in the tumor of the MKN-28 models. **e**. Percentage of T cells in the PB of the MKN-28 models and representative FACS plots of the i.v group and the p.t group, respectively. **f**. Percentage of T cells in the spleen of the MKN-28 models and representative FACS plots of the i.v group and the p.t group, respectively. Error bars denote the s.e.m., and the results were compared with one-way ANOVA. *p < 0.05; **p < 0.01; ***p < 0.001

Having verified their cytokine release, activation, and cytolytic capacity in vitro, we then sought to evaluate the therapeutic efficacy of anti-PSCA CAR-T cells in vivo by establishing subcutaneous xenograft gastric cancer cell lines in NSI mice. BGC-823 cells were first transplanted, and mice were administered GFP-T cells and anti-PSCA CAR-T cells when Tumor nodules were palpable (Fig. 4a). A previous study validated that peritumoral delivery of CAR-T cells demonstrated improved efficacy compared to intravenous delivery in xenograft models targeting mesothelin [20]. Thus, we used both delivery strategies for each kind of CAR-T cell to further ascertain this phenomenon in our study. Tumor volume was measured twice a week. Compared to the mock group, the GFP-T cell-treated groups, and even the group treated with intravenous anti-PSCA CAR-T cells, the group treated with peritumoral anti-PSCA CAR-T cells showed significant antitumor efficacy (Fig. 4b, c). We also detected the percentage of T cell infiltration in the tumor, peripheral blood (PB), and spleen (Fig. 4d, e, f). Then, to further validate the cytotoxicity of anti-PSCA CAR-T cells in vivo, we established another mouse model by subcutaneously transplanting MKN-28 tumor cells and infused CAR-T cell at a more challenging size of ~ 100 mm3, simulating late-stage gastric cancer (Fig. 5a). Anti-CD19 CAR-T cells, universally recognized to eliminate CD19-expressing targets, were used as controls to obviate concerns about the CAR alone. Despite the fact that the heavier tumor burden slightly blunted the CAR-T cell effects, anti-PSCA CAR-T cells still displayed robust antineoplastic capacity when delivered peritumorally (Fig. 5b, c). However, anti-PSCA CAR-T cells transmitted by intravenous injection showed no significant therapeutic ability against PSCA-expressing tumor masses. To address this issue, we examined the T cell level in different organs after sacrificing the mice. The T cell infiltration proportion in tumors was not significantly different between the anti-PSCA CAR-T cell-treated groups and the control groups (Fig. 5d), regardless of the administration patterns. Only a small proportion of T cells could be detected in tumor tissues. Further analysis of the peripheral blood (PB) and spleen revealed an increased percentage of T cells in the intravenous injection group compared with the peritumorally injected group (Fig. 5e, f), which is slightly different from the performance seen in the BGC-823 model (Fig. 4e, f). In summary, our data demonstrate the remarkable antitumor efficacy of anti-PSCA CAR-T cells in vivo when they are infused peritumorally.

## Discussion

Being the third leading cause of global cancer mortality [22], gastric cancer has gradually imposed a considerable health burden around the world [17, 23]. Traditional treatments such as surgery are only available for early-stage gastric cancer [24], while chemotherapy is usually not recommended for elderly patients [25], and most patients are not particularly sensitive to these drugs. Targeted therapy has emerged as a promising treatment strategy in recent years, with trastuzumab, targeted at human epidermal growth factor receptor-2 (HER2), and ramucirumab, targeted at vascular endothelial growth factor receptor-2 (VEGFR-2), being approved by the Food and Drug Administration [26, 27]. However, HER2 is only overexpressed in 13–22% of patients with gastric cancer [28, 29], underlining the necessity of discovering new treatment approaches.

The advent of CAR-T cell therapy triggered a paradigm shift in cancer immunotherapy [30]. The application of CAR-T cells in hematological malignancies has achieved compelling success [31], sparking considerable interest in in-depth research for this field. Until now, the effects obtained for the treatment of solid tumors have been relatively less encouraging [32]. A potential caveat is posed by the fact that solid tumors are heterogeneous, especially gastric cancer [33]. The lack of truly tumor-specific target antigens is one of the major obstacles. To provide more possibilities for clinical efficacy, we conducted a tentative experiment targeting PSCA in gastric cancer. We constructed a third-generation CAR composed of two costimulatory molecules as previously described [20] and confirmed the potential of PSCA as an ideal antigen for CAR-T cell therapy in gastric cancer. The third-generation CAR we applied herein composed of two costimulatory molecules, CD28 and DAP10. It has been corroborated by our lab that DAP10, the NKG2D associated adaptor protein, is a perfect costimulatory molecule for CAR-T therapy. Incorporation DAP10 in the second-generation CAR-T cells could enhance anti-tumor capacity both in vitro and in vivo [34]. We assayed canonical T cell activation and memory related markers after coculturing anti-PSCA CAR-T cells with target cell lines. Anti-PSCA CAR-T cells exhibited upregulated activation markers and cytokine production profiles in an antigen-dependent manner. Importantly, we demonstrated that anti-PSCA CAR-T cells delivered by peritumoral injection successfully stunted tumor progression in vivo. However, anti-PSCA CAR-T cells given intravenously failed to have any therapeutic effects. Although CAR-T cells given intravenously demonstrated slightly augmented T cell level in the tumor, peripheral blood, and spleen in MKN-28 cell line based mouse model, there were no statistically significant differences. The absolute number of CAR-T cells remains low, especially within the tumor. Hence, we did not pay much attention to this phenomenon. We then analyzed T cell population in the tumor, the data showed that CAR positive T cell percentage moderately increased compared to that of the pre-infused CAR-T cells. It is also possible that there were free PSCA in the blood that masked the CAR-T cells. However, it has been previously reported that CARs can dissociate from dying tumor cells even more rapidly than TCRs [35]. Hence, we did not make it the primary cause of the impaired CAR-T cell cytotoxicity. Much more effort might be taken to investigate whether it is a general phenomenon or a rare case and the mechanism hidden behind. Statistical significance was observed in the tumor weight between the peritumoral injection group and the intravenous injection group in the BGC-823 model, while no differences were seen in the MKN-28 model. Presumably, the augmented peripheral blood and spleen T cell levels in the peritumoral injection group might have contributed to this deviation. Consistently, these data unveiled another challenge impeding CAR-T cell application in solid tumors, that is, the difficulty of T cells in migrating to and physically infiltrating into the tumor [36]. As previously reported, much of the current literature on CAR-T cell therapy for solid tumors pays particular attention to T cell trafficking. Kheng Newick (2016) noted that blocking protein kinase A (PKA) localization successfully augmented CAR-T cell trafficking and antitumor efficacy [37]. Along the same lines, Qun Gao (2019) found that DOC induced CD8+ T cell recruitment to the tumor microenvironment by enhancing the secretion of HMGB1 and CXCL11 [38], underlining another view that successful trafficking relies on an appropriate match between the chemokine receptors on T cells and the chemokines secreted by the tumors [39]. The evidence reviewed here seems to suggest a combination strategy, including anti-PSCA CAR-T cells and chemical drugs.

Altogether, our findings corroborated the feasibility and efficacy of anti-PSCA CAR-T cells against gastric cancer, thus providing a new solution for interpatient and intratumor heterogeneity.

## Conclusion

This study identified PSCA as an ideal target for CAR-T cell therapy in GC and achieved impressive efficacy when using third-generation CAR-T cells for GC treatment. The results of this research reveal that anti-PSCA CAR-T cells can specifically eliminate target cells, both ex vivo and in vivo, implicating the potential of anti-PSCA CAR-T cells in treating GC patients in the clinic.

## Data Availability

All data generated or analyzed are available from the corresponding author on reasonable request.

## Abbreviations

ALL: Acute lymphoblastic leukemia
CAR: Chimeric antigen receptor
E:T: Effector to target
ELISA: Enzyme-linked immune absorbance assay
GC: Gastric cancer
GM-CSF: Granulocyte-macrophage colony-stimulating factor
i.p.: Intraperitoneally
i.v.: Intravenous
IFNγ: Interferon-γ
IL-2: Interleukin-2
p.t.: Peritumoral
p.t.: Peritumoral
PB: Peripheral blood
PBMC: Peripheral blood mononuclear cells mononuclear cells
PBMCs: Peripheral blood
PSCA: Prostate stem cell antigen
s.c.: Subcutaneous
scFv: Single-chain variable fragment
